# Treatment of Secondary Hemophagocytic Lymphohistiocytosis Associated With Diffuse Large B-cell Lymphoma Using Loncastuximab Tesirine As Lymphoma-Directed Therapy

**DOI:** 10.7759/cureus.70450

**Published:** 2024-09-29

**Authors:** Timothy Schieber, Jordan Snyder, Forat Lutfi

**Affiliations:** 1 Hematology/Oncology, The University of Kansas Cancer Center, Kansas City, USA; 2 Hematology, The University of Kansas Cancer Center, Kansas City, USA

**Keywords:** dlbcl, ferritin, hlh, loncastuximab, malignancy, secondary, treatment

## Abstract

Three critically ill, chemotherapy-refractory patients with diffuse large B-cell lymphoma (DLBCL) received loncastuximab tesirine in conjunction with standard therapies for secondary malignancy-associated hemophagocytic lymphohistiocytosis (Mal-HLH). All patients were treated inpatient, with one requiring intubation on the day of administration. Each patient had an H-score >238, indicating a >98% probability of HLH. A significant reduction in ferritin levels was observed in two patients, and one patient achieved a complete response (CR). Loncastuximab tesirine demonstrated promise in managing Mal-HLH where previous treatments had failed. This study suggests that loncastuximab tesirine exhibits favorable activity and should be considered a valuable addition to the treatment options for Mal-HLH driven by DLBCL.

## Introduction

Hemophagocytic lymphohistiocytosis (HLH) is a rare hyperinflammatory condition associated with multiorgan dysfunction and a high incidence of mortality. While primary HLH generally affects pediatric patients due to inherited genetic mutations, secondary HLH occurs in adult patients and is often propagated by malignancy, infection, or autoimmune disorders [1,2]. The incidence of HLH from malignancy has been reported from 1% to 2.8% in patients with lymphoma. Presentation often occurs with the new diagnosis of cancer or progression of cancer on therapy with associated laboratory abnormalities [1]. Although controversy remains on the diagnosis of HLH in adults as primary and secondary HLH are induced via different mechanisms, current guidelines still recommend utilizing the HLH-2004 diagnostic criteria for both [1,2]. Additional studies have validated the high specificity and sensitivity of the HScore and ferritin greater than 10,000 ng/mL alone for the diagnosis of HLH [3,4]. Monitoring for HLH improvement often includes clinical status and labs utilized for the diagnosis of HLH. Ferritin decreases of less than 50% from baseline are associated with a 17-fold increase in mortality compared to greater than 96% decreases validating the ferritin trend as one of the most important monitoring parameters for HLH [5].

Early treatment recommendations come from the HLH-94 protocol with additional updates in HLH-2004, which utilized corticosteroids and etoposide as standard HLH-directed therapy in pediatric patients with primary HLH [5]. Additional agents such as anakinra, ruxolitinib, intravenous immunoglobulin, and emapalumab among others have been reported [1]. There is limited data on the treatment of malignancy-associated HLH (Mal-HLH) though consensus recommendations extrapolate standard HLH agents used in the HLH-94 protocol along with malignancy-directed therapy [6]. Treatment reviews of Mal-HLH from diffuse large B-cell lymphoma (DLBCL) include etoposide-containing CHOP-like regimens alongside standard HLH therapy [1,6]. Omission of lymphoma-directed therapy has shown a 100% mortality rate showing lymphoma-directed therapy is the cornerstone of Mal-HLH treatment [7,8]. Treatment of chemotherapy-refractory DLBCL has been revolutionized recently with the utilization of chimeric antigen receptor T-cell therapy (CAR-T), bispecific T-cell engagers (BITE), antibody-drug conjugates, and lymphoma-directed monoclonal antibodies among other options [9-17]. No literature regarding the treatment of acute Mal-HLH from DLBCL with these new agents alongside HLH-directed therapy was identified prior to this study. Herein, we report our experience of the treatment of critically ill hospitalized patients with Mal-HLH from DLBCL with loncastuximab tesirine plus standard HLH-directed therapy in chemotherapy-refractory patients.

## Case presentation

Three patients were retrospectively identified from 2023 through May 2024 who received inpatient administration of loncastuximab tesirine for Mal-HLH. All patients had an H-score of >238 conferring a >98% probability of HLH (Table 1). The key outcomes evaluated were ferritin response defined as greater than 50% reduction and disease response to loncastuximab tesirine.

**Table 1:** Patient Characteristics and Treatment Response DLBCL, diffuse large B-cell lymphoma; non-GCB, non-germinal center B-cell subtype; R-CHOP, rituximab, cyclophosphamide, doxorubicin, vincristine, prednisone; RICE, rituximab, ifosfamide, carboplatin, etoposide; R-HyperCVAD (ARM B), methotrexate, cytarabine; Pola-BR, polatuzumab, bendamustine, rituximab; CNS, central nervous system; PET, positron emission tomography; ICU, intensive care unit; AST, aspartate aminotransferase; CR, complete response; N/A, not assessed

Patient	Age	Sex	Diagnosis	Treatment History	Response to Last Treatment	CNS Disease	HLH-2004 Diagnostic Criteria Met	Hscore (Probability of HLH)	Baseline Ferritin (ng/mL)	Level of Care Day of Loncastuximab Administration	Adjunct Therapy (Days Before/After Loncastuximab Administration)	Fibrinogen Resolution (>250 g/L)	AST Resolution (<50 units/L)	Ferritin Response (>50% Reduction)	Disease Response
1	55	M	DLBCL	RCHOP x3 cycles; RICE x1 cycle	Progressive disease (cycle 1, day 19)	Yes	Yes	>250 (>99%)	>30,000	ICU	Steroids per HLH-94 (started day -5)	Yes	Yes	Yes	CR
2	63	F	DLBCL (non-GCB)	R-HyperCVAD (ARM B) x3 cycles; R-CHOP x1 cycle; consolidative BCNU/thiotepa autologous transplant	Progressive disease (day +34)	Yes	Yes	238 (98-99%)	24,326	Intubated in ICU	Steroids per HLH-94 (started day -3), Anakinra (day -3 to day 5), Ruxolitinib (day -3 to day 5), Tocilizumab (day -1), IV Immunoglobulin (day -1), Emapalumab (day 1), Intrathecal methotrexate (day 3)	No	Yes	Yes	N/A
3	68	M	DLBCL (non-GCB)	R-CHOP x6 cycles; RICE x2 cycles; CD19 autologous CAR-T clinical trial; Pola-BR x 6 cycles; CD22 autologous CAR-T clinical trial; Glofitamab	CD22 CAR-T: PR (day +30 PET) followed by progressive disease (day +90 PET); Glofitamab full treatment dose never received due to hospital admission	No	Yes	>250 (>99%)	26,763	Medical oncology floor	Steroids per HLH-94 (started day -4), Anakinra (day 3 to day 6)	No	No	No	N/A

**Figure 1:**
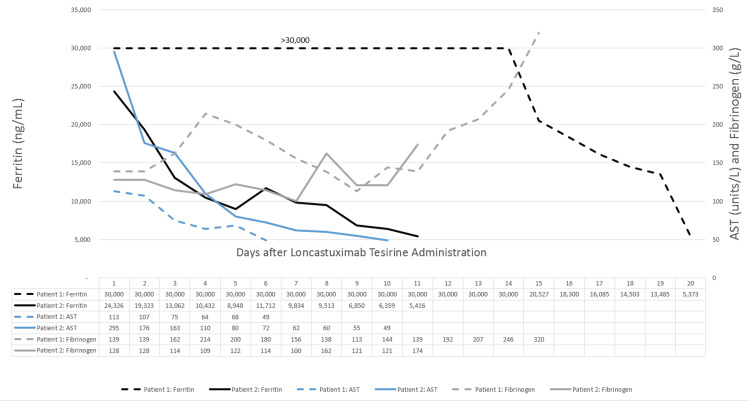
HLH Laboratory Trends After Loncastuximab Tesirine Administration in Patients 1 and 2 HLH, hemophagocytic lymphohistiocytosis; AST, aspartate aminotransferase

Patient 1 presented 19 days after salvage chemotherapy with recurrent Mal-HLH progression of disease (POD) noted on positron emission tomography-computed tomography (PET/CT). They were transferred to the intensive care unit (ICU), and steroids were initiated followed by loncastuximab tesirine. Symptomatic central nervous system disease was noted on the day of loncastuximab tesirine administration thus high-dose methotrexate was added eight days after administration. The patient rapidly improved and was discharged after methotrexate clearance. The ferritin level was not quantified above 30,000 ng/mL making it unclear the true peak and onset of ferritin reduction. The patient had an 82% reduction of ferritin from 30,000 ng/mL with a rapid reduction noted at day 14 consistent with a fast onset of inflammatory response (Figure 1). Twenty-nine days after loncastuximab tesirine a complete response (CR) was noted by PET/CT. The goal was to bridge the patient to CAR-T or allogeneic hematopoietic stem cell transplant (HSCT) though the patient later developed gastric perforation with infection leading to their death weeks after the resolution of Mal-HLH.

Patient 2 presented to the hospital on day +28 of consolidative autologous HSCT with a new-onset fever. She was transferred to the ICU on day +33 with a diagnosis of Mal-HLH, and POD was confirmed with DLBCL in the bone marrow on day +34. Due to neurocognitive decline associated with Mal-HLH, the patient was intubated for airway protection and given loncastuximab tesirine on day +36 of autologous HSCT. Due to the severity of the patient's condition and rapid decline, multiple HLH-directed therapies were utilized (Table 1). The patient had a 78% reduction from peak ferritin levels 11 days after loncastuximab tesirine administration, and the onset of ferritin decline occurred one day after administration (Figure 1). Ten days after loncastuximab tesirine administration, the patient clinically improved and was extubated with resolution of neurotoxicity. The patient elected to stop further aggressive care and was discharged.

Patient 3 presented to the hospital with an infection after previously noted POD (Table 1). While admitted further, POD occurred with new-onset Mal-HLH. Loncastuximab tesirine was administered along with HLH-directed therapy (Table 1). HLH was not attributed to prior CAR-T exposure. No signs of clinical or laboratory response were noted. The patient was transferred to inpatient hospice due to refractory Mal-HLH.

The treatment of Mal-HLH with loncastuximab tesirine plus HLH-directed therapy resulted in ferritin response in 66.7% (N=2/3) of patients and disease response with a CR in 33.3% (N=1/3) of patients. Patient 2 had clinical response leading to improved neurotoxicity and extubation though disease evaluation was not performed after the patient declined further treatment.

## Discussion

Loncastuximab tesirine was selected for the treatment of chemotherapy refractory Mal-HLH over other agents due to multiple theorized advantages. These three patients were deemed refractory to traditional chemotherapy thus previously studied CHOP-like regimens for Mal-HLH were unlikely to add efficacy [7,8]. Loncastuximab tesirine is a CD19-targeted antibody-drug conjugate that allows the delivery of a targeted payload of high-potency chemotherapy with a known bystander effect to overcome traditional chemotherapy resistance. The onset of loncastuximab tesirine is reported at 1.3 months though this only coincides with the first disease evaluation in the trial, not the true onset. Due to the mechanism and chemotherapy component, it was theorized to have a more rapid onset than other agents, which was shown by the rapid ferritin response with patients 1 and 2 [17,18]. Response rates within multiple subgroups and in the real-world setting with loncastuximab tesirine also remain clinically significant [13,19,20]. Finally, loncastuximab tesirine has not been demonstrated to increase the probability of loss of CD19 antigen expression; therefore, it may be an appropriate bridge to CAR-T therapy or allogeneic transplant, which was desired in patients 1 and 2 [18].

Polatuzumab, bendamustine, and rituximab would theoretically be a good option though bendamustine was not expected to add efficacy in the chemotherapy refractory setting. Response rates with polatuzumab monotherapy or polatuzumab plus rituximab were reported at 20% as opposed to 42% with the three-drug combination [14]. Tafasitamab plus lenalidomide have reported poor outcomes in retrospective cohorts and the antibody-mediated cellular toxicity along with lenalidomide-associated cytopenia were deemed less desirable for acute Mal-HLH [15,16]. CAR-T and BITE therapy are highly effective though they are not options acutely due to the treatment schedule with BITEs and logistics of CAR-T products [9-11]. This led to the selection of loncastuximab tesirine.

## Conclusions

To our knowledge, this is the first case series describing the successful treatment of acute chemotherapy refractory Mal-HLH from DLBCL with loncastuximab tesirine as lymphoma-directed therapy. While none of the three patients were successfully bridged to potentially curative therapy, we believe loncastuximab tersine has favorable activity and should be the latest addition in the armamentarium in DLBCL-driven Mal-HLH. Other reports of Mal-HLH treatment have only utilized traditional chemotherapy regimens highlighting the need for further studies in the treatment of Mal-HLH in chemotherapy refractory patients.
